# Transylvania University

**Published:** 1849-07

**Authors:** 


### TRANSYLVANIA UNIVERSITY.

Prof. Annan has been transferred to the chair of Theory and Practice, lately vacated by Prof. Bartlett, and Prof. William M. Bolling, late of the Memphis Medical College, has been elected to the chair of Obstetrics and the Diseases of Women and Children, in Transylvania University. Prof. B. is well known to the readers of American medical journals as the author of many able papers on a variety of subjects. He is a writer of unquestioned talents, industry and learning, and although not so well known as a lecturer, the experience of one winter in Memphis authorizes the belief that he will not be less successful in that line. His reputation in the South is high, and his election will strengthen the school in that quarter. We tender to Prof. Bolling our cordial wishes for his success.

